# Bilateral congenital alveolar synechiae—a rare cause of trismus

**DOI:** 10.1186/s40902-016-0056-2

**Published:** 2016-02-19

**Authors:** Smriti Panda, Kapil Sikka, Jyotsna Punj, Suresh C. Sharma

**Affiliations:** 1 Department of ENT, Teaching Block, All India Institute of Medical Sciences, 4th Floor, East Ansari Nagar, New Delhi, 110029 India; 2 Department of Anesthesiology, Teaching Block, All India Institute of Medical Sciences, 5th Floor, East Ansari Nagar, New Delhi, 110029 India

## Abstract

Congenital alveolar synechiae is a rare anomaly mostly presenting in association with cleft palate. Owing to reduced mouth opening, feeding difficulties, and compromised airway in extreme cases along with presentation in early neonatal period, these patients present unique challenges to the surgeon as well as the anesthetist. Here, we discuss the surgical and anesthetic management of this entity in a 12-month-old female child.

## Background

Bilateral congenital alveolar synechiae is a rare clinical entity frequently described in association with other craniofacial anomalies [1]. This condition is recognized in the early neonatal period due to feeding difficulties, restricted mouth opening, and in extreme cases, presenting with respiratory distress. Early surgical intervention is therefore warranted to address the neonates’ nutritional needs and optimal growth. The surgical, anesthetic, and airway challenges that these patients present with have been reviewed in this case report.

## Case presentation

A 1-year-old female was referred to our department with complaints of decreased mouth opening since birth. The child was born out of a non-consanguineous marriage. The mother had a full-term vaginal delivery. Birth weight was 2.5 kg. Apgar scores were unknown. The perinatal period was uneventful. The clinician attending the child, however, made note of the reduced mouth opening and the presence of bands adhering the upper and lower alveolus. Family history was also not contributory. The child attained age appropriate milestones and had no other systemic manifestations. The patient had no feeding or breathing difficulties and sought medical help for reduced mouth opening at the age of 12 months.

**Fig. 1:**
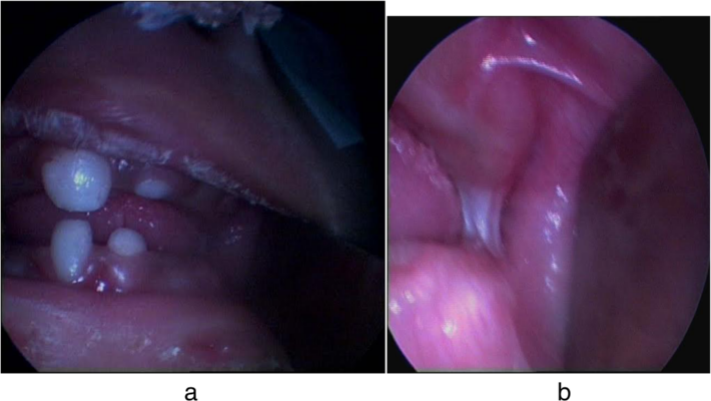
Preoperative findings. **a** Preoperative mouth opening and presence of a single central incisor. **b** Endoscopic view showing presence of synechiae on the left side

On examination, there were no dysmorphic features. Cardiovascular and neurological assessments were normal. No other musculoskeletal anomalies were found. Detailed oral cavity examination could not be performed due to trismus. Evaluation of oral cavity was performed with a nasal endoscope and two fibrous bands could be identified between upper and lower alveolus of thickness of 3 to 4 mm approximately, about 3 mm from the oral commissure on the left and 5 mm from the commissure on the right (Fig 1). The patient also had a concomitant bifid uvula. Bony ankylosis of temporomandibular joint was ruled out on computed tomography.

### Operative details

**Fig. 2:**
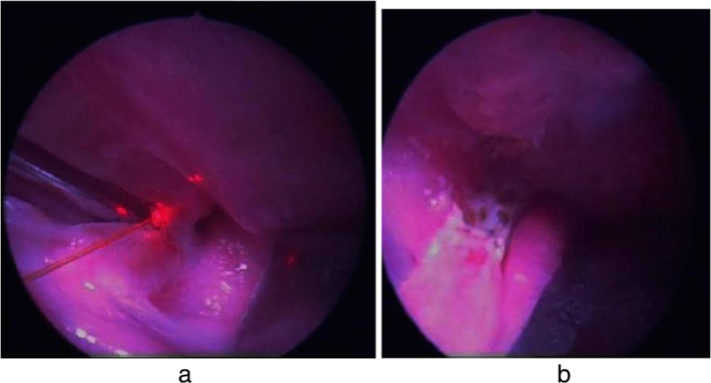
Intraoperative image. **a** Diode laser being used to release synechiae. **b** Minimal damage to mucosa after application of laser with limited charring

**Fig. 3:**
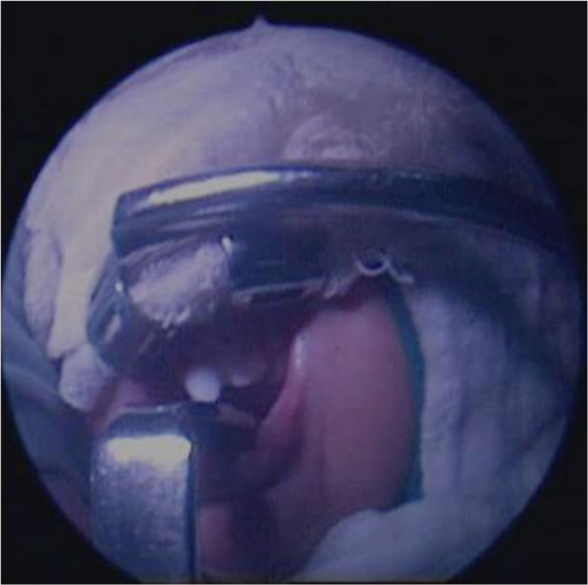
Postoperative mouth opening. After synechiae release, Boyle Davis mouth gag could be inserted

Patient was planned for laser release under general anesthesia. Owing to trismus, fiberoptic naso tracheal intubation was planned with an uncuffed 4-mm endotracheal tube. The child was premedicated with xylometazoline nasal drop (Otrivin 0.1 % *w*/*w*, Novartis, India) and injection glycopyrrolate (Vagolate 0.2 mg, 0.2 mg/ml, Abbott Health Care Pvt Ltd, India). Nasotracheal intubation was done with a size 4 uncuffed endotracheal tube with flexible endoscopic guidance, and after confirmation of correct position of the endotracheal tube, muscle relaxation was achieved with atracurium (Artacil—100, 0.9 % /10 mg /10 ml, Neon Laboratories Ltd, India). Nasopharynx and larynx were found to be normal and devoid of any fibrous bands or synechiae. Under endoscopic guidance, the oral synechiae were released with diode laser, 980 nm (ARC Diode Laser, FOX 10 W, Nurnberg, Germany) pulsed mode at 7.5 W power (Fig. 2). After release, a Boyle Davis mouth gag could be applied and a 3-cm mouth opening could be achieved (Fig. 3).

Patient had an uneventful postoperative recovery. Oral feeds were initiated immediately after recovery from anesthesia and was tolerated well by the patient.

### Discussion

Congenital intraoral synechiae is a rare congenital anomaly with approximately 60 cases documented in the literature [2]. It rarely presents in isolation. Of the 50 cases reported by Gartlan [3], only seven patients presented with isolated alveolar fusion. Associated anomalies include Van der Woude syndrome, popliteal pterygium syndrome, and orofacial digital syndrome [3]. The most common association is with cleft palate (cleft palate lateral synechiae syndrome) [1, 3].

These patients present in the early neonatal period due to restriction of mouth opening interfering with feeding and airway [1, 2, 4]. Our patient, however, presented late due to absence of gross craniofacial anomalies, absence of severe trismus owing to posterior location of the bands. Bifid uvula was the only concomitant anomaly found.

Various theories have been put forth to explain the association between cleft palate and intraoral synechiae. The most convincing is the sequence of cleft palate predisposing to increased mucosal contact between tongue and developing alveolus [1]. Other possible mechanisms like persistence of buccopharyngeal membrane and local ischemia of amniotic bands causing pressure on the first branchial arch also explain the rare occurrence of isolated alveolar synechiae. Airway management presents unique challenges to the anesthesia team. Blind nasal intubation was attempted in the past [5]. Tracheostomy is, generally, reserved as a last resort in these patients with only one article quoting the need for tracheostomy in a 38-week-old male infant presenting with marked desaturations [3]. Nasotracheal intubation with flexible fiberoptic bronchoscope as performed in our case is considered the gold standard [1, 5].

Treatment consists of dividing the bands as early as the child’s general condition permits to optimize feeding, craniofacial development, and prevent fibrous ankylosis due to disuse. Diode laser has been used in our patient to minimize blood loss, post operative pain, prevent collateral damage, and hence hasten the healing process and prevent reformation of the adhesions.

## Conclusions

Trismus in a neonate presents unique challenges to the treating physician for airway and feeding management. Timely referral to oral and maxillofacial center with good anesthetic expertise can circumvent the problems of failure to thrive and poor development. Flexible fiberoptic intubation can avoid the additional morbidity caused by tracheostomy in such young infants. The use of diode laser not only reduced surgical time but also played a significant role in expediting healing process and helped in early initiation of oral feeds.

## Consent

Written informed consent was obtained from the patient for publication of this case report and any accompanying images. A copy of the written consent is available for review by the Editor-in-Chief of this journal.
